# Genome-wide in-silico analysis of ethylene biosynthesis gene family in *Musa acuminata* L. and their response under nutrient stress

**DOI:** 10.1038/s41598-023-51075-3

**Published:** 2024-01-04

**Authors:** Nosheen Tabassum, Muhammad Shafiq, Sameen Fatima, Sana Tahir, Bushra Tabassum, Qurban Ali, Muhammad Arshad Javed

**Affiliations:** 1 Department of Horticulture, Faculty of Agricultural Sciences, University of the Punjab New Campus, Lahore, Pakistan; 2 School of Biological Sciences, University of the Punjab New Campus, Lahore, Pakistan; 3 Department of Plant Breeding and Genetics, Faculty of Agricultural Sciences, University of the Punjab New Campus, Lahore, Pakistan

**Keywords:** Computational biology and bioinformatics, Plant sciences

## Abstract

Ethylene is a gaseous phytohormone involved in plants’ growth and developmental processes, including seed germination, root initiation, fruit ripening, flower and leaf senescence, abscission, and stress responses. Ethylene biosynthesis (EB) gene analysis in response to nitrogen (N) and potassium (K) stress has not yet been conducted in *Musa acuminata* (banana) roots. The genome mining of banana (*Musa acuminata* L.) revealed 14 putative 1-aminocyclopropane-1-carboxylate synthase (*ACS*), 10 1-aminocyclopropane-1-carboxylate oxidase (*ACO*), and 3 Ethylene overproducer 1 (*ETO1*) genes. *ACS, ACO,* and *ETO1* proteins possessed amino acid residues ranging from 422–684, 636–2670, and 893–969, respectively, with molecular weight (Mw) ranging from 4.93–7.55 kD, 10.1–8.3 kD and 10.1–10.78 kD. The number of introns present in *ACS, ACO,* and *ETO1* gene sequences ranges from 0–14, 1–6, and 0–6, respectively. The *cis*-regulatory element analysis revealed the presence of light-responsive, abscisic acid, seed regulation, auxin-responsive, gibberellin element, endosperm-specific, anoxic inducibility, low-temperature responsiveness, salicylic acid responsiveness, meristem-specific and stress-responsive elements. Comprehensive phylogenetic analyses *ACS*, *ACO,* and *ETO1* genes of Banana with *Arabidopsis thaliana* revealed several orthologs and paralogs assisting in understanding the putative functions of these genes. The expression profile of *Musa acuminata* genes in root under normal and low levels of nitrogen and potassium shows that *MaACS*14 and *MaACO*6 expressed highly at normal nitrogen supply. *MaACS*1 expression was significantly upregulated at low potassium levels, whereas, *MaACO*6 gene expression was significantly downregulated. The functional divergence and site-specific selective pressures on specific gene sequences of banana have been investigated. The bioinformatics-based genome-wide assessment of the family of banana attempted in the present study could be a significant step for deciphering novel *ACS*, *ACO,* and *ETO1* genes based on genome-wide expression profiling.

## Introduction

As a gaseous phytohormone, ethylene is produced in most plant tissues. It is important in regulating plant growth and developmental processes, including seed germination, root initiation, root gravitropism, fruit ripening, flower and leaf senescence, abscission, and stress responses. In plant tissues, ethylene production is typically low but increases at developmental stages such as ripening and senescence and in response to mechanical and environmental stresses^1^. Nutrient stress is a complex phenomenon that leads to low or high availability of nutrients to the plant^2^. Different genes are also involved in plant responses due to nutrient stresses^3^. Hence, to understand the nutrient stress response mechanism of *Musa acuminata* in roots, it is important to identify and analyze the genes involved in ethylene biosynthesis. Furthermore, the ethylene biosynthesis (EB) genes in response to nitrogen (N) and potassium (K) stress have not yet been analyzed in *Musa acuminata* (banana) roots.

Ethylene also acts as an important modulator and inducer of plant aging^4^. Ethylene is biosynthesized from Methionine pathways in higher plants in which *ACS* and ACC oxidase (*ACO*) catalyze the reactions from *S*-adenosylmethionine (SAM) to 1-aminocyclopropane-1-carboxylate (ACC) and ACC to ethylene, respectively^5,6^. This ethylene biosynthesis pathway has been well explained and mapped in the *Arabidopsis thaliana*^7^. Using advanced molecular biology techniques, various plant species have been used to isolate cDNA and genomic clones for both enzymes, encoded by multigene families^8–13^. These cDNA clones have been used to characterize the expression of individual members in various tissues and in response to recognized ethylene biosynthesis-inducing stimuli^7^. In Arabidopsis, *ACS*2, *ACS*4 to *ACS*9, and *ACS*11 form functional homodimers, whereas unfunctional homodimer forms in *ACS*1. *ACS*3 is a pseudogene. Whereas *ACS*10 and *ACS*12 encode aminotransferase. All genes play a specific role in the plant during growth and development. It was studied that *AtACS*5 gene had the highest promoter activity in grown seedlings (2 weeks old). And this gene was localized at the reproductive stage from areoles where *AtACS*4 and *AtACS*7 were present in both veins and areoles^14^. Ethylene gas is a plant hormone and critical growth regulator. It synthesizes in response to biotic and abiotic stresses^15–19^. It also influences plant growth and developmental processes like germination, leaf and flower senescence and abscission, fruit ripening, and nodulation^19,20^. In most plant tissues, the level of *ACS* activity is almost directly proportional to the level of ethylene production^21^. The synthesis of ethylene involves simple yet highly regulated steps. *ACS*’s conversion of S-adenosyl methionine to ACC is a rate-limiting step in ethylene biosynthesis. ACC is converted to ethylene by ACC oxidase (*ACO*). 1-Aminocyclopropane-1-carboxylate oxidase (*ACO*), is an O_2_-activating ascorbate-dependent nonheme iron enzyme, which involves in the catalyses of ACC in the ethylene biosynthetic pathway and converts 1-aminocyclopropane-1-carboxylic acid (ACC) to ethylene^22^. An ethylene overproducer mutation, ETO1, negatively regulates *ACS* and ethylene production. *ETO1* regulates the stability of 1-aminocyclopropane-1-carboxylate synthase (*ACS*) enzymes. It can also act as substrate-specific adapter connecting *ACS* enzymes like *ACS*5 to ubiquitin ligase complexes, leading to proteasomal degradation of *ACS* enzyme^23,24^. A constitutive triple-response phenotype in Arabidopsis was used to identify three mutants that were affected in the regulation of ethylene biosynthesis due to ethylene overproduction^25,26^. The post-transcriptional regulation of 1-aminocyclopropane-1-carboxylic acid synthase (*ACS*) has been affected by the Arabidopsis ethylene-overproducing mutants *ETO*1, *ETO*2, and *ETO*3. *ETO*2 and *ETO*3 are dominant mutations, but *ETO*1 is inherited as a recessive mutation. The 12-amino-acid C-terminal region of *ACS*5 is predicted to be disrupted by the 1-bp insertion that causes the *ETO*2 mutation. Since the steady-state level of *ACS*5 mRNA is not increased by the *ETO*2 mutation, the mutation likely works post-transcriptionally^27^.

A comparative study of 1-aminocyclopropane-1-carboxylic acid synthase (*ACS*), 1-aminocyclopropane-1-carboxylate oxidase (*ACO*), and Ethylene overproducer 1 (*ETO1*) genes from ethylene biosynthesis pathway in *M. acuminata* was performed with *A*. *thaliana* would provide a starting point for understanding how the *M. acuminata* gene family response under different stresses. Genome-wide analysis identified ACS, ACO, and ETO genes in M. acuminata. *M. acuminata* gene’s structures, functions, and conserved motifs were compared with *A*. *thaliana*’s *ACS, ACO,* and *ETO* gene to investigate their response under nitrogen (N) and potassium (k) stress. Furthermore, the expression of *ACS, ACO,* and *ETO* genes in *M. acuminata* in response to nitrogen (N) and potassium (k) stress was investigated. The present findings suggest the roles of *ACS, ACO, and ETO* genes in mediating abiotic stress and provide valuable information for further study on the function of *ACS, ACO,* and *ETO* genes in the growth, development, and stress responses of *M. acuminata.*

## Materials and methods

### Database search and retrieval of sequence

377 Amino acid (AA) sequence of PLP_aminotran (CL0061)^28^ present in *AtACS* (Accession no. NP_191710.1), *ACO* (Accession no. NP_179549.1) and full-length AA sequence of *ETO1* (Accession no. NP_190745.6) retrieved from the *Arabidopsis thaliana* proteome database at NCBI Gene bank and was used for the identification of *ACS, ACO* and *ETO1* proteins-encoding genes in the banana proteome database at Phytozome https://phytozome.jgi.doe.gov/pz/portal.html using BLAST-P program^29^. The incorrect and redundant predicted sequences were manually removed, and then all putative *MaACS, MaACO,* and *MaETO* genes were further verified using Pfam database. The retrieved amino acid sequences were subjected to NCBI CDD (Conserved Domain Database) (http://www.ncbi.nlm.nih.gov/Structure/cdd/wrpsb.cgi)^30^ with the default parameters.

### Determination of physical characteristics of banana proteins

Amino acid length, molecular weight, and theoretical pI of *MaACS, MaACO,* and *MaETO* proteins were predicted using ProtParam tool (http://web.expasy.org/protparam/)^31^. The information for gene IDs, chromosomal position, and gene and protein sequence was retrieved from Phytozome. All genes were renamed according to the order of their physical position. Subcellular localization of *MaACS, MaACO,* and *MaETO* was predicted using the online tool WoLF PSORT (https://wolfpsort.hgc.jp/)^32^.

### Multiple sequence alignment and phylogenetic analysis

The amino acid sequences of *ACS,* proteins from banana, and arabidopsis were aligned using Clustal W version 2.1^33,34^. The phylogenetic tree was constructed with MEGAx.0 using a neighbor-joining (NJ) method^35^. The bootstrap values were calculated for 1000 iterations. Similar protein alignment and phylogenetics analysis methods were used for *ETO* and *ACO* proteins. 14 *MaACS* genes and 12 *Arabidopsis thaliana* protein sequences were used for phylogenetic analysis. Similarly, for *ACO*, 10 *MaACO* genes and 5 *Arabidopsis thaliana* protein sequences, and for *ETO*, 3 *MaETO* genes and 5 *Arabidopsis thaliana* protein sequences were used.

### Gene structure analysis and conserved motifs recognition

The genomic and coding sequences of identified genes were retrieved from the database to investigate the intron/exon arrangement of banana *ACS*, *ACO,* and *ETO* genes. These sequences were further used to draw the gene structure using Gene Structure Display Server (GSDS v2.0^36^) (available at http://gsds.cbi.pku.edu.cn/). Multiple EM for Motif Elicitation (MEME) programs (http://meme.nbcr.net/meme/)^37^ was used to analyze the concluded protein sequences of the banana *ACS, ACO,* and *ETO*-1 with a maximum number of motif set as 20.

### Gene duplication and calculation of Ka and Ks substitution rates

Putative Gene pair was generated using the knowledge obtained from phylogenetic, motif, and domain analysis of *M. acuminata ACS, ACO,* and *ETO1* genes. These gene pairs were used to calculate the Ka and Ks values through tbtools^38^. The CDS, protein sequence, and gene pair of *MaACS, MaACO,* and *MaETO* were utilized. Using Ka/Ks ratio, molecular evolutionary rates of each gene pair were calculated. The Ka/Ks ratio less than 1 indicates the viability of purifying selection; however, Ka/Ks ratio greater than 1 indicates positive selection, and Ka/Ks = 1 indicates neutral selection^39,40^. Using the formula “t = Ks/2λ”, with λ (6.05 × 10^−9^), gene pair divergence was estimated to represent neutral substitution. The *MaACS, MaACO,* and *MaETO* like genes were mapped on scaffold using TBtool, and duplicated genes were connected on scaffold using red lines.

### Promoter analysis

To analyze the organ-specific expression profile of *MaACS, MaACO* and *MaETO* at various development stages, the *cis*-regulatory element evaluation of recognized banana *ACS, ACO,* and *ETO1* genes was performed by recovering 1000 base pairs sequence upstream from the starting site of banana genomic sequences (promoter region) using the Phytozome database. An ample number of cis-regulatory elements were analyzed in all the *Musa acuminata* 14 *ACS, 10 ACO,* and 3 *ETO* genes by employing the PlantCare database (http://bioinformatics.psb.ugent.be/webtools/plantcare/html/)^41^.

### Expression analysis

For expression profiling, Reads Per Kilo bases per Million mapped reads (RPKM) values from RNA-seq data were log^2^ transformed, and the p-value was calculated using *t* test. Expression patterns with hierarchical clustering are displayed in Heatmap Illustrator in TBtools^38^.

### Putative microRNA target sites analysis

The micro-RNA (miRNA) datasets of *Musa acuminata* were retrieved from a knowledge-based database called Plant miRNA Encyclopedia (PmiREN, http://www.pmiren.com/)^42^. There, to find out the miRNAs that target the *MaACS, MaACO,* and *MaETO* genes, CDS sequences of all *MaACS*, *MaACO,* and *MaETO* genes were searched for the complementary sequences of miRNAs with the help of psRNA Target (https://plantgrn.noble.org/psRNATarget/analysis?function=3)^43^ with default parameters.

### Ethical approval

It has been confirmed that the experimental samples of plants, including the collection of plant material, complied with relevant institutional, national, and international guidelines and legislation with appropriate permissions from Authorities of Department of Horticulture, Faculty of Agricultural Sciences, University of the Punjab New campus, Lahore, Pakistan.

## Results

### Identification of the *ACS*, *ACO* and* ETO1* genes in banana

*ACS*, *ACO,* and *ETO* genes, involved in the ethylene biosynthesis pathway, were detected in Banana Genome database. Accordingly, the initial analysis predicted 26, 18, and 8 protein sequences of *ACS*, *ACO,* and *ETO*, respectively. The proteins encoded by the same gene isoforms and proteins containing truncated *ACS, ACO,* and *ETO* DNA-binding domains were manually removed. Finally, 14 *MaACS,* 10 *MaACO,* and 3 *MaETO* non-redundant genes were identified and used for further analysis. The number of *ACS* genes was more than that of *ACO* and *ETO*.

The studied genes encoded proteins ranging from 443 to 2670 amino acids in length; *MaACS*3 was the smallest protein, whereas *MaACO*6 was the most significant protein. Molecular weight varied between 10.16 and 83.3. Isoelectric points ranged from 8.47 (*MaACS*6) to 4.79 (*MaACO*7) (Tables 1, 2, 3).

**Table 1:** Information about 14 *MaACS* genes discovered from the genome of *M. acuminata*, their orthologue in *Arabidopsis* and their functional annotation.

*ACS* gene	Source accession no		Chromosome no	Chromosome location	Direction (bp)	mRNA	Size (AA)		pI	Mw (D)	Gene expression of banana in roots (Fig. 6)	Orthologue from model *Arabidopsis thaliana* and or other plants			
	Phytozome	Banana Genome Hub					Peptide	Genome				Gene ID	Accession no	Annotation/function in plants	References
*MaACS*1	GSMUA_Achr4G29150_001	Ma04_g35640	4	27240988..27243490	Reverse	1461	486	2504	7.97	54,964.73	It showed high expression in roots during low potassium stress	*AtACS2*	*NP_171655*	These enzymes catalyze the conversion of S-adenosyl-L-methionine (SAM) into 1-aminocyclopropane-1-carboxylate (ACC), a direct ethylene precursor	^44^
*MaACS*2	GSMUA_Achr4G24930_001	Ma04_g31490	4	24594460..24596343	Reverse	1275	481	1884	6.62	53,971.55	It did not show any expression in roots or leaves under N, K stress	*AtACS6*	*NP_192867*	It is a direct precursor of ethylene. Involved in bacterial flagellin-induced ethylene production	^45^
*MaACS*3	GSMUA_AchrUn_randomG12290_001	Ma09_g19150	Un-characterized	58535286..58538567	Forward	1443	443	3282	7.61	49,795.41	It requires a high potassium level to express itself in roots	*AtACS6*	*NP_192867*	Involved in bacterial flagellin-induced ethylene production	^45^
*MaACS*4	GSMUA_Achr2G08600_001	Ma02_g10500	2	12544533..12546250	Reverse	1341	463	1718	7.81	51,966.40	It expressed moderately in roots during low nitrogen level	*AtACS9*	*NP_190539*	*ACS* proteins' stability and regulation of such stability play a central role in ethylene biosynthesis	^44^
*MaACS*5	GSMUA_Achr3G25830_001	Ma03_g27050	3	25860557..25862163	Forward	1299	449	1607	8.23	50,282.53	It did not express in roots or leaves either in any nitrogen or potassium level	*AtACS8*	*NP_195491*	Promotes early biosynthesis of ethylene that elicits plant immunity in Arabidopsis	^42^
*MaACS*6	GSMUA_Achr5G08880_001	Ma05_g08580	5	6453130..6454804	Forward	1401	466	1675	8.47	52,017.26	It did not express in roots or leaves either in any nitrogen or potassium level	*AtACS8*	*NP_195491*	Promotes early biosynthesis of ethylene that elicits plant immunity in Arabidopsis	^42^
*MaACS*7	GSMUA_Achr1G04420_001	Ma04_g24230	1	3704286..3705947	Forward	1305	462	1662	6.51	51,501.56	It did not express in roots or leaves either in any nitrogen or potassium level	*AtACS8*	*NP_195491*	Promotes early biosynthesis of ethylene that elicits plant immunity in Arabidopsis	^42^
*MaACS*8	GSMUA_Achr4G01340_001	Ma04_g01260	4	1083786..1085438	Forward	948	462	1653	7.58	51,938.54	It did not express in roots or leaves either in any nitrogen or potassium level	*AtACS8*	*NP_195491*	Promotes early biosynthesis of ethylene that elicits plant immunity in Arabidopsis	^42^
*MaACS*9	GSMUA_Achr1G14280_001	Ma01_g07800	1	10895407..10897053	Forward	1374	457	1647	7.15	51,130.24	It showed very slight expression in both low nitrogen and low potassium in roots	*AtACS9*	*NP_190539*	*ACS* proteins' stability and regulation of such stability play a central role in ethylene biosynthesis	^44^
*MaACS*10	GSMUA_Achr10G27570_001	Ma10_g27510	10	30681506..30683062	Forward	477	440	1557	5.27	49,310.04	It did not express in roots or leaves either in any nitrogen or potassium level	*AtACS7*	*NP_194350*	It is involved in root gravitropism in *Arabidopsis thaliana*	^46^
*MaACS*11	GSMUA_Achr4G30870_001	Ma04_g37400	4	28274327..28275910	Forward		422	1584	5.35	47,224.60	It did not express in roots or leaves either in any nitrogen or potassium level	*AtACS7*	*NP_194350*	It is involved in root gravitropism in *Arabidopsis thaliana*	^46^
*MaACS*12	GSMUA_Achr1G18880_001	Ma01_g12130	1	14122481..14126525	Forward	1524	507	4045	6.99	55,839.15	It did not express in roots or leaves either in any nitrogen or potassium level	*AtACS12*	*NP_001330155*	This gene encodes aminotransferases	^44^
*MaACS*13	GSMUA_Achr3G12300_001	Ma03_g12030	3	9096959..9105359	Reverse	1.1494 2. 486	684	8401	8.44	75,564.75	It did not express in roots or leaves either in any nitrogen or potassium level	*AtACS12*	*NP_001330155*	This gene encodes aminotransferases	^44^
*MaACS*14	GSMUA_Achr5G13730_001	Ma02_g09230	5	9876405..9880334	Forward	1464	503	6138	8.05	55,406.70	It expressed highly under low nitrogen or controlled nitrogen in roots and leaves both of plant	*AtACS12*	*NP_001330155*	This gene encodes aminotransferases	^44^

**Table 2:** Information about 10 *MaACO* genes discovered from the genome of *M. acuminata*, their orthologue in *Arabidopsis* and their functional annotation.

*ACO* gene	Source accession		Chromosome no	Chromosome location	Direction (bp)	Size (AA)	pI	M_w_ (D) Gene ID	Gene expression of banana in roots (Fig. 6)	Orthologue from model *Arabidopsis thaliana* and or other plants			
	Phytozome	Banana genome hub				Peptide				Gene ID	Accession no	Annotation/function in plants	References
Ma *ACO*1	GSMUA_Achr7T18420_001	Ma07_g9730	7	21215281..21216618	Forward	1338	4.99	110,033.70	It showed no expression in roots or leaves under N, K stress	*AtACO3*	*NP_171655*	Enzyme involved in the ethylene biosynthesis. May promote stem elongation by maximizing the extensibility of cells, possibly by activating ethylene biosynthesis, in response to very-long-chain fatty acids	^47^
*MaACO*2	GSMUA_Achr1T18250_001	Ma01_g11540	1	13635443..13636669	Forward	1227	4.97	101,618.97	It expressed moderately in roots during low potassium level	*AtACO2*	*NP_192867*	Required to mediate the 1-aminocyclopropane-1-carboxylic acid (ACC)-mediated reversion of the ABA-induced inhibition of seed germination via endosperm rupture	^47^
*MaACO*3	GSMUA_Achr10T16070_001	Ma10_g16100	10	23745515..23747172	Forward	1658	4.94	136,735.63	It expressed moderately high in roots during low nitrogen level	*AtACO4*	*NP_192867*	Activate ethylene biosynthesis in response to very-long-chain fatty acids	^47^
*MaACO*4	GSMUA_Achr6T12910_001	Ma06_g14390	6	8622468..8623805	Reverse	1338	5.00	109,682.80	It expressed moderately in roots during low potassium level	*AtACO4*	*NP_190539*	Enzyme involved in the ethylene biosynthesis. May promote stem elongation by maximizing the extensibility of cells, possibly by activating ethylene biosynthesis, in response to very-long-chain fatty acids	^47^
*MaACO*5	GSMUA_Achr6T00870_001	Ma06_g02600	6	598708..599734	Forward	1027	5.03	83,222.98	It expressed moderately in roots during low potassium and nitrogen level	*AtACO5*		Enzyme involved in ethylene biosynthesis	^47^
*MaACO*6	GSMUA_Achr5T09690_001	Ma05_g09360	5	6911660..6914329	Reverse	2670	4.86	221,359.93	It showed very slight expression in low nitrogen and low potassium in roots	*AtACO5*			
*MaACO*7	GSMUA_AchrUn_randomT20420_001	Ma06_g14370	Un	99012649..99015822	Forward	3074	4.79	252,277.03	It was not expressed in roots or leaves in any nitrogen or potassium level	*AtACO4*		Enzyme involved in the ethylene biosynthesis. May promote stem elongation by maximizing the extensibility of cells, possibly by activating ethylene biosynthesis, in response to very-long-chain fatty acids	^47^
*MaACO*8	GSMUA_AchrUn_randomT26960_001	Ma06_g14430	Un	132016604..132017996	Forward	1393	5.00	114,867.70	It showed moderate expression in both low nitrogen and low potassium in roots	*AtACO4*		Enzyme involved in the ethylene biosynthesis. May promote stem elongation by maximizing the IIextensibility cells	^47^
*MaACO*9	GSMUA_AchrUn_randomT20430_001	Ma06_g14420	Un	99027647..99028973	Forward	1327	4.92	108,789.51	It showed very slight expression in both low nitrogen and low potassium in roots	*AtACO4*		Activate ethylene biosynthesis in response to very-long-chain fatty acids	^47^
*MaACO*10	GSMUA_AchrUn_randomT28750_001	Ma0_g04770	Un	139442789..139443424	Forward	636	5.13	53,238.13	It did not express in roots or leaves either in any nitrogen or potassium level	*AtACO3*		Enzyme involved in the ethylene biosynthesis. May promote stem elongation by maximizing the extensibility of cells, possibly by activating ethylene biosynthesis, in response to very-long-chain fatty acids	^47^

**Table 3:** Information about 10 *MaACO* genes discovered from the genome of *M. acuminata.*

*ETO* gene	Source accession		Chromosome no	Chromosome location	Direction (bp)	Size (AA)		pI	M_w_ (D)
	Phytozome	Banana genome hub				Genomic	Peptide		
*MaETO*1	GSMUA_Achr6G32850_001	Ma06_g34510	6	32072728..32078672	Reverse	8518	969	6.02	107,866.66
*MaETO*2	GSMUA_Achr8G01920_001	Ma08_g01750	8	1432050..1436389	Reverse	5945	965	6.42	107,534.88
*MaETO*3	GSMUA_Achr4G21140_001	Ma04_g27750	4	21647264..21656370	Reverse	4340	893	5.78	101,212.79

### Comparative phylogenetic relatedness of banana *ACS*, *ACO* and *ETO1* gene family with Arabidopsis

**Figure 1:**
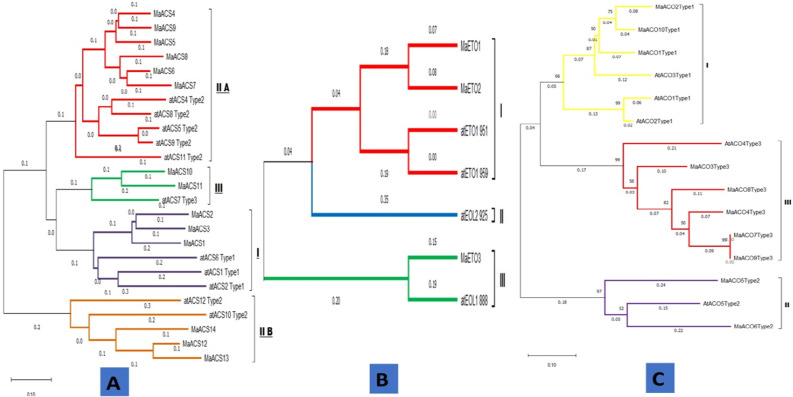
Phylogenetic analysis of *Musa acuminata* (*MaACS*) (**A**), *MaETO* (**B**) and *MaACO* (**C**) genes*.*

To investigate the evolutionary relationships between *MaACS, MaACO* and *MaETO1* TFs and *Arabidopsis thaliana,* a neighbor-joining (NJ) phylogenetic tree was constructed by aligning their full-length protein sequences. The results showed that 14 *MaACS* proteins were distributed among 3 groups named I, II A, II B, and III (Table S1 and Fig. 1A).

Group I consisted of 3 Arabidopsis proteins while 3 belong to banana *MaACS1, MaACS3*. II-A group consisted of 5 Arabidopsis *AtACS*4, *AtACS*5, *AtACS*8, *AtACS*9, *AtACS*11 and 6 genes of *MaACS*4, *MaACS*5, *MaACS*6, *MaACS*7, *MaACS*8, *MaACS*9. II-B Group contained 2 *Arabidopsis thaliana* proteins *AtACS*12, *AtACS*10, while *Musa acuminata* consisted of *MaACS*12, *MaACS*13, *MaACS*14. Group III contained only 1 Arabidopsis protein *AtACS*7, whereas banana included *MaACS*10 and *MaACS*11.

The results for *ETO1* depicted that 3 *MaETO1* proteins were distributed among 3 groups named I, II, and III (Table S2 and Fig. 1B).

Group I consisted of 2 *Arabidopsis* *thaliana* *AtETO*1_951, *AtETO*1_959 proteins while 2 belong to banana *MaETO1, MaETO2*. II groups consist of only 1 Arabidopsis AtEOL2_925, and 0 banana genes. Group III contained only 1 Arabidopsis protein *AtEOL*1_888, whereas banana included *MaETO*3. According to *ACO* results, the division of *MaACO* into groups represents that group I contains 3 *Arabidopsis thaliana* proteins *AtACO*1, *AtACO*2, *AtACO*3, and 3 *Musa acuminata* genes, i.e. *MaACO1, MaACO2, MaACO10.* Group II consisted of 1 *Arabidopsis thaliana* protein *AtACO*5 and 2 *Musa acuminata* genes *MaACO5 and MaACO6. Similarly, group* III contain 1 *Arabidopsis thaliana* protein *AtACO*4 and 5 *Musa acuminata* genes *MaACO3, MaACO4, MaACO7, MaACO8, MaACO9* (Table S3 and Fig. 1C).

Proteins of common clade usually seem to show similarity in structure and functioning. So, all the *ACS*, *ACO,* and *ETO1* like proteins of similar Clades may have similar structures and functions.

### Gene structures and recognition of conserved motifs and domain

**Figure 2:**
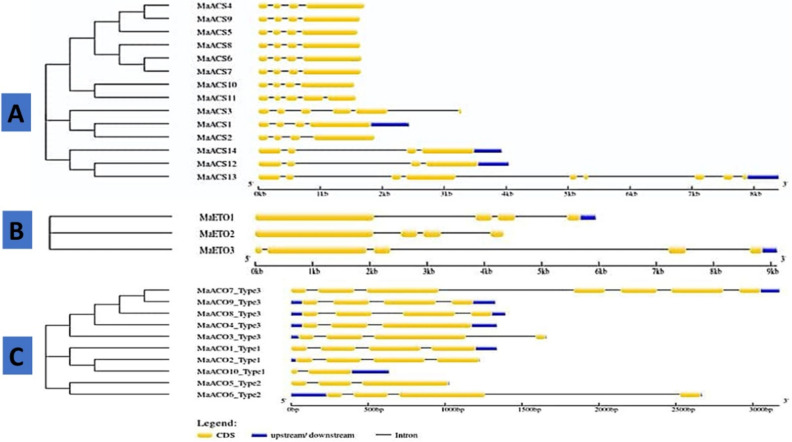
Phylogenetic relationship and gene structure of ACS, ETO and ACO genes from *M*. *acuminata*.

The organization of exon and intron helps verify the evolutionary relationship between genes or organisms^48^. Their distribution patterns and numbers are an evolutionary mark for a gene family. A comprehensive demonstration of Banana genes' exon–intron structures and phylogenetic revealed that the gene structure pattern was consistent with the phylogenetic analysis. The number of introns varied from 3 to 8 in *MaACS* gene, and no gene in *MaACS* is intron-less (Fig. 2A, Table S4).

In Group I, *MaACS*1 possesses 3 introns and 4 exons. Group IIA comprises of *MaACS*2 gene which has 3 introns and 4 exons. In group IIB, *MaACS*3 contain 5 introns and 6 exons, while *MaACS*4, *MaACS*5, *MaACS*6, *MaACS*7 genes contain 4 exons and 3 introns. In group III, *MaACS*11 gene contains 5 exons and 4 intron and *MaACS*13 gene has 9 exons and 8 introns, while, *MaACS*8, *MaACS*9, *MaACS*10, *MaACS*12 and *MaACS*14 consists of 4 exons and 3 introns.

According to Fig. 2B and Table S5, group I contain *MaETO1* and *MaETO*2 genes consisting of 4 exons and 3 introns, while *MaETO*3 in group III has 4 exons and 3 introns. Similarly, *MaACO*1 and *MaACO*2 of group I contain 4 exons and 3 introns, while *MaACO*10 of group I have 2 exons and 1 intron. *MaACO*5 of group II comprises 2 introns and 3 exons, while *MaACO*6 contains 4 exons and 3 introns. *MaACO*3, *MaACO*8, and *MaACO*9 of group III contain 4 exons and 3 introns, while *MaACO4* has 3 exons and 2 introns, and *MaACO7* consists of 7 exons and 6 introns (Fig. 2C, Table S6).

**Figure 3:**
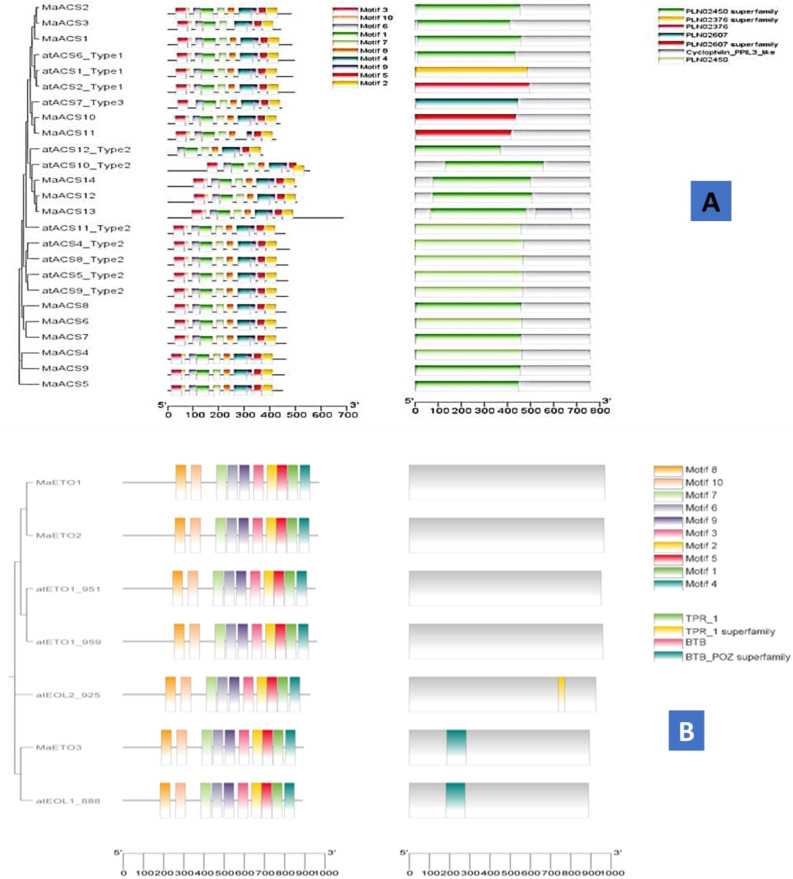

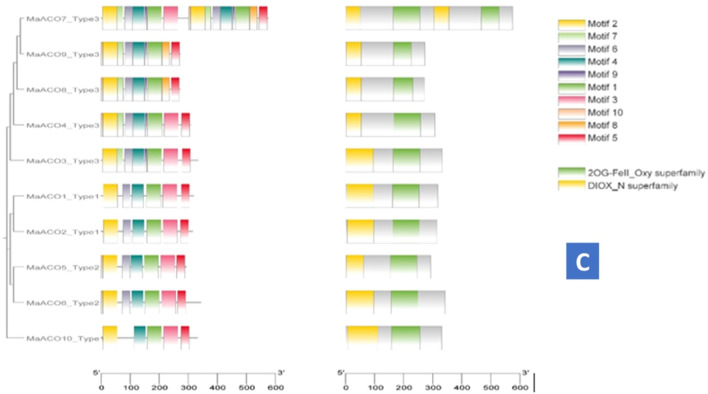
The distribution of 10 motifs present in *MaACS* (**A**), *MaETO* (**B**) and *MaACO* (**C**) protein of banana.

The conserved motif analysis also verified the classification of *MaACS* genes. All *MaACS* protein sequences were loaded into the MEME analysis tool to identify the conserved motifs. As a result, twenty conserved motifs were observed, which were statistically significant with *E*-values less than 1 × 10^−40^ (Fig. 3). The motifs of *MaACS* proteins identified by MEME were between 15–50 amino acids in length. Motif 1, Motif 2, Motif 5, Motif 6, Motif 7 and Motif 9 are common in all groups. Group I and Group IIA had similar motif patterns. Motif 3 is also common in all groups except in *AtACS*-12_Type 2 gene of group IIB. All groups (I, IIA, IIB and III) contain M-4 and M-8 other than Group III (*MaACS11*) and G-I (*MaACS3*), respectively. Meanwhile, IIB group members have relatively complex motif patterns compared with Group IIA (Fig. 3A).

In *MaETO* proteins, all the motifs are common in all groups. Motif pattern is also the same in all groups. Figure 3B shows that *MaETO* gene structures are similar to the corresponding *AtETO* gene structures. TPR-1 domain is only present in *AtETO* genes. BTB_POZ domain is only present in *MaETO3* genes, similar to *atEOL-1* genes.

Motif analysis of *MaACO* genes shows that motif 3 is only present in *MaACO7, MaACO8 MaACO9* of G-3. Motif 7 is only present in G-3 (*MaACO3, MaACO4, MaACO7, MaACO8, MaACO9*).* MaACO10* contains only 5 motifs i-e. 1, 2, 3, 4 and 5. *MaACO7* has repeats of motifs and domains. Both 2OG-Fell_Oxy and DIOX_N domains are present in all *MaACO* genes* (*Fig. 3C)*.*

### Gene duplication of banana *ACS*, *ACO* and *ETO*1 genes

**Figure 4:**
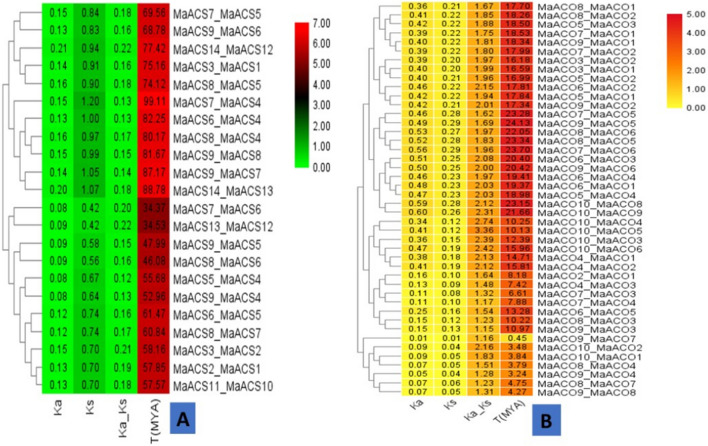
Time of gene duplication estimated for different paralogous pairs of *MaACS* (**A**) and *MaACO* (**B**) genes based on Ks and Ka values.

The date of duplication of the gene was also estimated through MEGA-X using pairwise alignment that provided Ks and Ka values and then Ka/Ks was calculated manually (Fig. 4). Ks depicts the number of synonymous substitutions per synonymous site, whereas Ka shows the number of nonsynonymous substitutions per nonsynonymous site and the ratio of nonsynonymous (Ka) versus synonymous (Ks) mutation was represented by Ka/Ks. The speculative date for gene duplication of the paralogous group *MaACS*7_*MaACS*4 was calculated to be 99.11Mya, while for the other remaining 21 *ACS* paralogous pairs, the segmental duplication date of *M. acuminata* was estimated in the range from 46.08 to 88.78 Mya for paralogous pairs 15 and 11. All the paralogous groups in *M. acuminata* had a Ka/Ks ratio greater than 0.12, suggesting the possibility of considerable functional divergence after the duplication process (Fig. 4A, Table S7).

The date of gene duplication for *ETO* of *M. acuminata* was calculated 17.26 Mya for paralogous group *MaETO2_MaETO1* (Table S8). Similarly, the gene duplication date for *MaACO* was calculated in 44 paralogous pairs. The segmental duplication date of banana was estimated from 0.45 to 24.13 Mya for paralogous pairs 38 and 14. In all paralogous groups of *MaACO* the Ka/Ks ratio is greater than 1.15 (Fig. 4B, Table S9).

### Analysis of *cis*-regulatory elements

**Figure 5:**
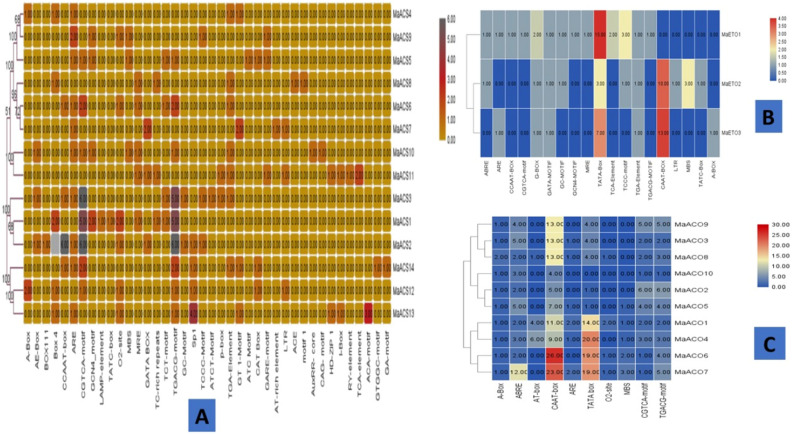
Different *cis*-acting elements in putative *MaACS* (**A**), *MaETO* (**B**) and* MaACO* (**C**) promoters which are associated with abiotic stresses, hormone responses, growth and development.

The spatial–temporal transcriptomic expression of genes is affected by the presence and organization of various *cis*-regulatory elements at the binding site of transcription factors on the promoter region. In-silico analysis of cis-regulatory elements can be employed to evaluate the putative functions of genes. Cis-regulatory elements related to vital physiological processes such as response to light, seed-specific, endosperm-specific, hormone-specific, meristem-specific, and stress were observed (Fig. 5). Mainly, 9 out of 14 *MaACS*, all 3 *MaETO* and 5 out of 10 *MaACO* genes possess element involved in light responsiveness, 6 *MaACS* genes possess a fragment of a conserved DNA module that takes part in light responsiveness, and 9 *MaACS*, 2 *MaETO* and All 10 *MaACO* elements involved in the abscisic acid response.1 *MaACS* and 1 *MaETO* gene possess elements involved in salicylic acid responsiveness, while 3 *MaACS* genes showed elements that show response in defense and stress, and 3 *MaACS* and 10 *MaACO* genes are related to meristem expression, 3 *MaACS* and 1 *MaETO* gene possess factors involved in low-temperature responsiveness, 4 *MaACS*, and 3 *MaETO* genes possess Auxin-responsive element, 2 *MaACS*, and 2 *MaETO* genes showed element specific to anoxic inducibility, 1 *MaACS* genes possess elements specific to seed regulation, 4 *MaACS* genes contain elements involved in endosperm expression**,** 6 *MaACS*, and 1 *MaETO* gene possess elements involved in element involved in gibberellin response, 6 *MaACO* genes essential for the anaerobic induction and all 10 *MaACO* genes involved in *MeJA-*responsiveness. The cis-regulatory elements identified among 14 *ACS*, 3 *ETO,* and 10 *MaACO* genes of banana and their functional annotation are shown in (Fig. 5, Tables S10, S11 and S12).

### General miRNA expression dataset of *Musa acuminata*

**Figure 6:**
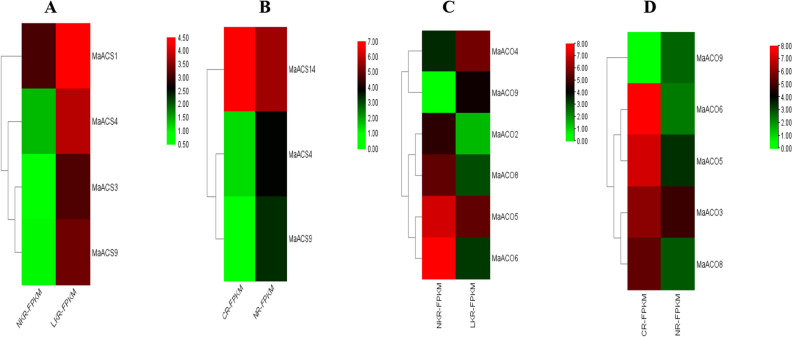
The heat map shows the expression profile of the *M. acuminata ACS* (**B**) and *ACO* (**D**) genes in root under the normal level of nitrogen (CR) and low level of nitrogen (NR) and *ACS* (**A**) and *ACO* (**C**) genes in roots under the normal level of potassium and low level of potassium in different organs. The expression levels of *MaACS* and *MaACO* genes are revealed by different colors, which increase from green to red.

Heat map for the expression profile of *Musa acuminata* genes in root under normal and low nitrogen and potassium shows that *MaACS*14 and *MaACO*6 expressed highly at a normal nitrogen supply level. Still, they do not respond significantly at low nitrogen levels. *MaACO*5, *MaACO*3, *MaACO*8 show very slight expression at normal nitrogen levels, while *MaACS*14 express slightly at low nitrogen levels. On the other hand, *MaACS*1 expresses well at low levels of potassium, whereas *MaACO*6 gives a significant response at normal levels of potassium. *MaACS*4, *MaACO*4 and *MaACO*5 show slight behavior in response to low potassium. (Fig. 6). The expression of each banana gene in roots is explained in Table 1.

The mature miRNAs sequences were retrieved from Plant MicroRNA Encyclopedia database. Later, those miRNAs that could potentially target *MaACS* genes were identified with the help of the psRNA Target online tool (https://plantgrn.noble.org/psRNATarget/analysis). Consequently, 49 miRNAs were found, targeting 8 out of 14 *MaACS* genes. The remaining 6 *MaACS* genes were not targeted by any of these miRNAs (Table 4). The number of miRNAs targeting these genes varies from 1 to 20 miRNAs per *MaACS* gene. *MaACS* 4, 9, 13, 14 are the genes targeted by only 1 miRNAs. On the other hand, *MaACS* 14 is targeted by 2 miRNAs. 3 miRNAs target *MaACS 4*. None of the gathered miRNAs targeted the remaining 6 *MaACS* genes. So, this indicates that *MaACS* 4 is the gene targeted by the maximum number of miRNAs. While discussing ba sed on groups, Group II A was targeted the most by these miRNAs, which was targeted by 3 miRNAs. On the other hand, Group A was targeted by only 3 miRNAs, which is the least among all (Table 4, Table S13).

**Table 4:** miRNA targets prediction of *MaACS*(A), *MaACO*(B) and *MaETO*(C).

miRNA_Acc	Target_Acc	No. of target found	Function of miRNA
Mac-miR396	*MaACS*6	20	Induced by cold but greatly repressed by heat stress^49^
Mac-miR396	*MaACS*8	2	Induced by cold but greatly repressed by heat stress^49^
Mac-miR396	*MaACS*14	1	Induced by cold but greatly repressed by heat stress^49^ Regulates transition of Arabidopsis root stem cells by transit-amplifying cells to form a regulatory circuit by repressing GRF^50^
Mac-miRN2003	*MaACS*2	3	
Mac-miRN2003	*MaACS*3	3	
Mac-miR159	*MaACS*4	5	Induced by cold but greatly repressed by heat stress^49^
Mac-miR319	*MaACS*4	11	Induced by cold but not by dehydration, salinity, or ABA^51,52^
Mac-miRN2002	*MaACS*13	1	
Mac-miRN2002	*MaACS*14	1	
Mac-miRN1993	*MaACS*9	1	
Mac-miRN2010	*MaACS*4	1	
Mac-miR390	*MaACO*7	6	
Mac-miR390	*MaACO*9	6	
Mac-miR391	*MaACO*5	1	
Mac-miR172	*MaACO*5	17	An important role during the banana fruit ripening process^53^
Mac-miR827	*MaACO*6	1	
Mac-miR1999	*MaACO*2	1	
Mac-miR171	*MaETO*1	7	Inhibit translation in plants^54^
Mac-miR428	*MaETO*1	1	The second most prevalent miRNA in NC, neural and ectoderm tissue but was not expressed in blastula^55^
Mac-miR 2009	*MaETO*2	2	
Mac-miR528	*MaETO*2	1	Significantly downregulated under cold stress and it was found to target genes encoding polyphenol oxidase (PPO) and copper‐containing proteins^56^

In *MaACO*, there are total 32 miRNAs that targeted 6 *MaACO* genes out of 10. The number of targeting miRNAs varies from 1 to 17. *MaACO5* targeted 2 miRNAs, and the remaining 4 genes were targeted by 1 miRNA. So, this depicts that *MaACO5* is the gene targeted by a maximum number of miRNAs (Table 4, Table S14). In *ETO* of banana, there are two 2 targeted miRNA for both *MaETO1* and *MaETO2*. The total number of targets is 11, the maximum number of miRNA targets on the *MaETO1* gene (Table 4, Table S15).

## Discussion

The coordination of genes, hormones, and environmental factors made the ripening process successful^57^. Genes like *ACS, ACO* and *ETO* involved in ethylene biosynthesis pathway^5,6^ perform key role in ripening^58^. Banana genome database (https://phytozome-next.jgi.doe.gov/info/Macuminata_v1) implied to identify 14 *ACS* genes, 10 *ACO* genes and 3 *ETO* genes (Table 1) at the genome-wide level. The 14 banana *ACS* genes were classified into 4 subfamilies (Group I, IIA, IIB, III), whereas in *ETO* and *ACO*, there were divided into 3 subgroups (Group I, II, III) using the phylogenetic analysis (Fig. 1). The exon–intron structure and prediction can also be used as evidence for understanding the evolutionary relationships among genes or organisms^48,59,60^. The predicted exon–intron association revealed that all 14 genes have introns (Fig. 2A). The number of introns varied from 3 to 9 in *MaACS* gene (Fig. 2B), 3 to 4 in *MaETO* genes (Fig. 2B) and 1–6 in *MaACO* genes of banana (Fig. 2C). In general, it shows that banana *MaACS* genes in the same group share similar exon–intron structures (Fig. 2). Exon–intron having similar structures have also been noticed in Arabidopsis, rice and soybean^61,62^ which suggest that these structures are evolutionarily preserved.

Motif analysis shows that Motif 3 is common in all groups except in *AtACS*-12_Type 2 gene of group IIB. While, IIB group members have relatively complex motif patterns compared with Group IIA (Fig. 3A). Figure 3B shows that *MaETO* gene structures are similar to the corresponding *AtETO* gene structures. BTB_POZ domain is only present in *MaETO* gene that was vital for *AtETO* genes (Fig. 3B). Furthermore, *MaACO7* has motifs and PLN02299 domain repeats, similar to *CoACO*1 and *CoACO*2 in *Camellia oleifera*^63^. PLN02403 domain is only present in *MaACO5* and *MaACO6* (Fig. 3C). The distribution of motifs among *ACS* proteins (Fig. 3) indicates evolutionary and structural relationships as deduced by the phylogenetic tree^64–66^. The motif data analysis by MEME (Fig. 3), and domain analysis using NCBI CDD distinct motifs were identified that were differentially distributed among *MaACS* (Fig. 3). Meanwhile, at least one or two conservative motif types and spatial distributions in *MaACS* were present in the same subfamily while some differences were present, implying certain functional similarities of banana *ACS* members within the same subfamily. In addition, *MaACS* genes showed structural conservation in subfamilies and were consistent with other plants such as *Arabidopsis*, rice, cotton and chickpea^61,67–70^. In addition, as predicted by in silico analyses, 3 deduced *MaACS* harbored NLSs to help localize them to the nucleus, but subcellular localization analysis using online tool WoLF PSORT (https://wolfpsort.hgc.jp/), supposed nucleolus localization in almost all *MaACS* protein except *MaACS* 12 and *MaACS* 14.

The ratio of Ka/Ks provides an understanding of the selection pressure on substituting amino acids. Less than one ratio of Ka/Ks (Ka/Ks < 1) suggests the possibility of purifying selection, whereas more than one ratio of Ka/Ks (Ka/Ks > 1) suggests the likelihood of positive selection^39,71,72^. Generally, evaluation of selective pressure provides a particular lead for amino acid sequence altered in a protein and is also necessary for interpreting functional residues and protein shifts^73^. Ka/Ks ratios of the sequences from the different banana *MaACS* groups vary remarkably, while in *ETO*, only one gene showed Ka/Ks ratio. Despite the differences, all the estimated values of Ka/Ks were less than 1, suggesting that all the *ACS* sequences in each group undergo strong purifying selection pressure and positive selection might have acted on only a few sites during the process of evolution. In *MaACO*, all the estimated values of Ka/Ks which were more than one (> 1) shows the possibility of existence of significant positive selection after duplication (Fig. 4).

Heat map for the expression profile of *Musa acuminata* genes in root under normal and low nitrogen and potassium shows that *MaACS*14 and *MaACO*6 expressed highly at normal nitrogen supply. Whereas, *MaACS*1 represents well at low potassium levels, whereas *MaACO*6 gives a significant response at normal levels of potassium (Fig. 5). In contrast, *ACS* genes exhibited low expression under potassium and cadmium stress^74^. Similarly, ACS1, ACS4 and ACS7 expression were enhanced in response to UV treatment in tomatoes, while *ACS*3, *ACS*5 and *ACS*6 showed no variation^75^.

MicroRNAs are very important regulators of plants that regulate almost every biological process, ranging from growth and development to combating pathogens and maintaining proper internal conditions, as miRNA affects many genes of specified functions^43,76–79^. miRNAs are highly conserved among different species, as each microRNA performs a specific function, regardless of the type of species in which they were observed. In *Musa acuminata, MaACS4* is targeted by 3 miRNA i.e. Mac-miR159, Mac-miR319, and Mac-miRN2010, which are induced in cold stress and repressed in heat stress^49^, dehydration, salinity, or ABA^52^ and involved in root formation and cell elongation^51^. Mac-miR396 is an important miRNA that targets *MaACS*6, *MaACS*8 and *MaACS*14 that activate in cold stress and repress in heat stress^49^. It also regulates the transition of Arabidopsis root stem cells by transit-amplifying cells to form a regulatory circuit by repressing GRF^50^. *MaACS*14 is targeted by 2 miRNAs, Mac-miRN2002 and Mac-miR396, which are important for root stem cells in Arabidopsis^50^. *MaACO5* is targeted by 2 miRNA Mac-miR391 and Mac-miR172, which are involved in the ripening process of banana^53^. Mac-miR172 is a known MiRNA of banana^53^, has maximum targets on *MaACO5* and does not target any other protein in *MaACO*. In *MaETO*, both *MaETO*1 and *MaETO*2 are targeted by 2 miRNA. Mac-miR171 and Mac-miR428 both target on *MaETO*1, in which Mac-miR171 inhibits translation in plants^54^, and Mac-miR428 is expressed in neural and ectoderm tissue but was not expressed in blastula^55^. While Mac-miR N2009 and Mac-miR528 target *MaETO*2. Mac-miR528 targets many genes encoding copper‐containing proteins and polyphenol oxidase (PPO), and is downregulated in cold stress^56^. It is reported that three specialized miRNAs (miR173, miR390 and miR828) have been identified and well characterized in *Arabidopsis*^80–82^. Only miR390 is present in *MaACO7* and *MaACO9*. This might suggest that Mac-miR396 in *MaACS* genes are important for root cell elongation and maintaining the regulatory mechanism between root and stem.

## Conclusion

A comprehensive analysis of genes (*ACS*, *ACO*, and *ETO*1) involved in ethylene biosynthesis in the *Musa acuminata* (Banana) genome was discussed in this study. The 15 genes of *ACS*, 10 genes of *ACO* and 3 genes of *ETO*1 were categorised into subgroups. The structural and functional properties of each *MaACS, MaACO* and *MaETO* member were characterized under Nitrogen (N) and Potassium (K) stress in plant roots, where, *MaACO*6 expressed highly at both normal and low level of N and P, and *MaACS*14 expressed well at low nitrogen level, while, *MaACS*4, *MaACO*4 and *MaACO*5 responded to low potassium. Most genes were involved in root cell formation and maintaining the regulation mechanism between roots and stem, suggesting their role in plant root growth and development. The detailed computational inspection of Banana *ACS*, *ACO* and *ETO* proteins revealed in the current study might be selected for cloning purposes at the molecular level, portraying gene expression and studying their interaction with different transcription factors.

### Supplementary Information

Supplementary Tables.

## Data Availability

The datasets generated and/or analysed during the current study are available in the manuscript.

## Abbreviations

*ACS*: 1-Aminocyclopropane-1-carboxylate synthase
*ACO*: 1-Aminocyclopropane-1-carboxylate oxidase
*ETO*1: Ethylene overproducer 1
*M. acuminata*: *Musa acuminata*
pI: Isoelectronic point
Mw: Molecular weight
AA: Amino acid
kD: Kilodaltons
NJ: Neighbor Joining
Ka/Ks: The ratio of nonsynonymous substitutions per nonsynonymous site (Ka) to the number of synonymous substitutions per synonymous site (Ks).
N: Nitrogen
K: Potassium
