# Supplementary material for: Genome-wide in-silico analysis of ethylene biosynthesis gene family in Musa acuminata L. and their response under nutrient stress
Source: Sci Rep. 2024 Jan 4;14:558. doi: 10.1038/s41598-023-51075-3 (PMC10767074; doi:10.1038/s41598-023-51075-3)
Supplement: Supplementary file 1 — Supplementary Tables. [file 41598_2023_51075_MOESM1_ESM.docx]

**Table S1:** Banana ACS gene family distribution among groups based on phylogenetic analysis with Arabidopsis *ACS* member.

| Group | Number of ACS gene | | Gene id | |
| --- | --- | --- | --- | --- |
|  | AtACS | MaACS | AtACS | MaACS |
| I | 3 | 3 | AtACS1, AtACS2, AtACS6 | MaACS1, MaACS2, MaACS3 |
| II-A | 5 | 6 | AtACS4, AtACS5, AtACS8, AtACS9, AtACS11 | MaACS4, MaACS5, MaACS6, MaACS7, MaACS8, MaACS9 |
| II-B | 2 | 3 | AtACS12, AtACS10 | MaACS12, MaACS13, MaACS14 |
| III | 1 | 2 | AtACS7 | MaACS10, MaACS11 |

**Table S2:** Banana ETO gene family distribution among groups based on phylogenetic analysis with Arabidopsis *ETO* member.

| Group | Number of ETO gene | | Gene id | |
| --- | --- | --- | --- | --- |
|  | AtETO | MaETO | AtETO, AtEOLI, AtEOL2 | MaETO |
| I | 2 | 2 | AtETO1_951, AtETO1_959 | MaETO1, MaETO2 |
| II | 1 | 0 | AtEOL2_925 | No match |
| III | 1 | 1 | AtEOL1_888 | MaETO3 |

**Table S3:** Banana ACO gene family distribution among groups based on phylogenetic analysis with Arabidopsis *ACO* members.

| Group | Number of ACO gene | | Gene id | |
| --- | --- | --- | --- | --- |
|  | AtACO | MaACO | AtACO | MaACO |
| I | 3 | 3 | AtACO1, AtACO2, AtACO3 | MaACO1, MaACO2 , MaACO10 |
| III | 1 | 5 | AtACO4 | MaACO3 , MaACO4 , MaACO7, MaACO8 , MaACO9 |
| II | 1 | 2 | AtACO5 | MaACO5, MaACO6 |

**Table S4:** In M.accuminata predicted number of intron and exon in MaACS genes.

| **Group** | **GENE** | **Accession Number** | **Chromosome Location** | **Exon** | **Introns** |
| --- | --- | --- | --- | --- | --- |
| I | MaACS1 | GSMUA_Achr4G29150_001 | 27240988..2724349 | 4 | 3 |
| IIA | MaACS2 | GSMUA_Achr4G24930_001 | 24594460..24596343 | 4 | 3 |
| IIB | MaACS3 | GSMUA_AchrUn_randomG12290_001 | 58535286..58538567 | 6 | 5 |
| III | MaACS4 | GSMUA_Achr2G08600_001 | 12544533..12546250 | 4 | 3 |
|  | MaACS5 | GSMUA_Achr3G25830_001 | 25860557..25862163 | 4 | 3 |
|  | MaACS6 | GSMUA_Achr5G08880_001 | 6453130..6454804 | 4 | 3 |
|  | MaACS7 | GSMUA_Achr1G04420_001 | 3704286..3705947 | 4 | 3 |
|  | MaACS8 | GSMUA_Achr4G01340_001 | 1083786..1085438 | 4 | 3 |
|  | MaACS9 | GSMUA_Achr1G14280_001 | 10895407..10897053 | 4 | 3 |
|  | MaACS10 | GSMUA_Achr10G27570_001 | 30681506..30683062 | 4 | 3 |
|  | MaACS11 | GSMUA_Achr4G30870_001 | 28274327..28275910 | 5 | 4 |
|  | MaACS12 | GSMUA_Achr1G18880_001 | 14122481..14126525 | 4 | 3 |
|  | MaACS13 | GSMUA_Achr3G12300_001 | 9096959..9105359 | 9 | 8 |
|  | MaACS14 | GSMUA_Achr5G13730_001 | 9876405..9880334 | 4 | 3 |

**Table S5:** In M.accuminata predicted number of intron and exon in MaETO genes.

| **Group** | **GENE** | **Accession Number** | **Chromosome Location** | **Exon** | **Introns** |
| --- | --- | --- | --- | --- | --- |
| I | MaETO1 | GSMUA_Achr6G32850_001 | 32072728..32078672 | 4 | 3 |
| I | METO2 | GSMUA_Achr8G01920_001 | 1432050..1436389 | 4 | 3 |
| III | MaETO3 | GSMUA_Achr4G21140_001 | 21647264..21656370 | 5 | 4 |

**Table S6:** In *M.accuminata* predicted number of intron and exon in MaACO genes

| **Group** | **GENE** | **Accession Number** | **Chromosome Location** | **Exon** | **Introns** |
| --- | --- | --- | --- | --- | --- |
| I | MaACO1 | GSMUA_Achr7T18420_001 | 21215281..21216618 | 4 | 3 |
| I | MaACO2 | GSMUA_Achr1T18250_001 | 13635443..13636669 | 4 | 3 |
| III | MaAC03 | GSMUA_Achr10T16070_001 | 23745515..23747172 | 4 | 3 |
| III | MaACO4 | GSMUA_Achr6T12910_001 | 8622468..8623805 | 3 | 2 |
| II | MaACO5 | GSMUA_Achr6T00870_001 | 598708..599734 | 3 | 2 |
| II | MaACO6 | GSMUA_Achr5T09690_001 | 6911660..6914329 | 4 | 3 |
| III | MaACO7 | GSMUA_AchrUn_randomT20420_001 | 99012649..99015822 | 7 | 6 |
| III | MaACO8 | GSMUA_AchrUn_randomT26960_001 | 132016604..132017996 | 4 | 3 |
| III | MaACO9 | GSMUA_AchrUn_randomT20430_001 | 99027647..99028973 | 4 | 3 |
| I | MaACO10 | GSMUA_AchrUn_randomT28750_001 | 139442789..139443424 | 2 | 1 |

**Table S7:** Duplicated ACS Genes and their dates of duplication in M. acuminata

| Seq_1 | Ka | Ks | Ka/Ks | T(MYA) |
| --- | --- | --- | --- | --- |
| MaACS2_MaACS1 | 0.134923 | 0.700022 | 0.192741 | 5.79E+13 |
| MaACS3_MaACS1 | 0.141978 | 0.909386 | 0.156126 | 7.52E+13 |
| MaACS3_MaACS2 | 0.146003 | 0.703747 | 0.207465 | 5.82E+13 |
| MaACS5_MaACS4 | 0.080924 | 0.673743 | 0.120112 | 5.57E+13 |
| MaACS6_MaACS4 | 0.125642 | 0.995192 | 0.126249 | 8.22E+13 |
| MaACS7_MaACS4 | 0.152642 | 1.199265 | 0.12728 | 9.91E+13 |
| MaACS8_MaACS4 | 0.162051 | 0.969997 | 0.167064 | 8.02E+13 |
| MaACS9_MaACS4 | 0.080273 | 0.64084 | 0.125262 | 5.30E+13 |
| MaACS6_MaACS5 | 0.120969 | 0.7438 | 0.162636 | 6.15E+13 |
| MaACS7_MaACS5 | 0.152587 | 0.841658 | 0.181294 | 6.96E+13 |
| MaACS8_MaACS5 | 0.157378 | 0.89686 | 0.175476 | 7.41E+13 |
| MaACS9_MaACS5 | 0.08805 | 0.580646 | 0.151641 | 4.80E+13 |
| MaACS7_MaACS6 | 0.083461 | 0.415899 | 0.200677 | 3.44E+13 |
| MaACS8_MaACS6 | 0.089244 | 0.557605 | 0.160049 | 4.61E+13 |
| MaACS9_MaACS6 | 0.129828 | 0.832293 | 0.155989 | 6.88E+13 |
| MaACS8_MaACS7 | 0.123138 | 0.736165 | 0.167269 | 6.08E+13 |
| MaACS9_MaACS7 | 0.144554 | 1.054785 | 0.137046 | 8.72E+13 |
| MaACS9_MaACS8 | 0.149832 | 0.988203 | 0.151621 | 8.17E+13 |
| MaACS11_MaACS10 | 0.127957 | 0.696596 | 0.183689 | 5.76E+13 |
| MaACS13_MaACS12 | 0.091529 | 0.417789 | 0.21908 | 3.45E+13 |
| MaACS14_MaACS12 | 0.210744 | 0.93678 | 0.224967 | 7.74E+13 |
| MaACS14_MaACS13 | 0.198526 | 1.07425 | 0.184804 | 8.88E+13 |

**Table S8**: Duplicated ETO-like Genes and their dates of duplication in M. acuminata

| Seq_1 | Ka | Ks | Ka/Ks | T(MYA) |
| --- | --- | --- | --- | --- |
| MaETO2_MaETO1 | 0.077923 | 0.515731 | 0.151092 | 4.26E+13 |

**Table S9**: Duplicated ACO-like Genes and their dates of duplication in M. acuminata

| Sequence | Ka | Ks | Ka_Ks | T(MYA) |
| --- | --- | --- | --- | --- |
| MaACO2_MaACO1 | 0.16259 | 0.098919 | 1.643673 | 8.17512407 |
| MaACO3_MaACO1 | 0.398501 | 0.200734 | 1.985218 | 16.58960185 |
| MaACO4_MaACO1 | 0.379038 | 0.177973 | 2.129746 | 14.7085392 |
| MaACO5_MaACO1 | 0.41918 | 0.215867 | 1.941843 | 17.84026287 |
| MaACO6_MaACO1 | 0.475321 | 0.234386 | 2.027945 | 19.37071503 |
| MaACO7_MaACO1 | 0.39294 | 0.224233 | 1.752375 | 18.53165755 |
| MaACO8_MaACO1 | 0.356668 | 0.214194 | 1.665166 | 17.70198208 |
| MaACO9_MaACO1 | 0.401522 | 0.221922 | 1.809294 | 18.34066153 |
| MaACO10_MaACO1 | 0.085239 | 0.046509 | 1.832761 | 3.843678105 |
| MaACO3_MaACO2 | 0.386122 | 0.195787 | 1.972158 | 16.18071172 |
| MaACO4_MaACO2 | 0.405288 | 0.191329 | 2.118275 | 15.8123493 |
| MaACO5_MaACO2 | 0.403183 | 0.20552 | 1.961774 | 16.9850953 |
| MaACO6_MaACO2 | 0.463195 | 0.215529 | 2.149109 | 17.81232182 |
| MaACO7_MaACO2 | 0.392282 | 0.217671 | 1.802176 | 17.98936544 |
| MaACO8_MaACO2 | 0.409659 | 0.220988 | 1.853762 | 18.26347573 |
| MaACO9_MaACO2 | 0.421152 | 0.209773 | 2.007654 | 17.33664623 |
| MaACO10_MaACO2 | 0.091228 | 0.042166 | 2.163573 | 3.484766085 |
| MaACO4_MaACO3 | 0.132855 | 0.089722 | 1.480742 | 7.415056235 |
| MaACO5_MaACO3 | 0.420562 | 0.22387 | 1.878603 | 18.50163152 |
| MaACO6_MaACO3 | 0.513071 | 0.24688 | 2.078222 | 20.40328104 |
| MaACO7_MaACO3 | 0.105932 | 0.080021 | 1.323807 | 6.613295859 |
| MaACO8_MaACO3 | 0.152722 | 0.123712 | 1.234495 | 10.22411145 |
| MaACO9_MaACO3 | 0.15321 | 0.132772 | 1.153938 | 10.97287753 |
| MaACO10_MaACO3 | 0.359038 | 0.149913 | 2.394966 | 12.38954476 |
| MaACO5_MaACO4 | 0.465326 | 0.229638 | 2.02635 | 18.9783247 |
| MaACO6_MaACO4 | 0.462706 | 0.234814 | 1.970519 | 19.40612971 |
| MaACO7_MaACO4 | 0.111249 | 0.095353 | 1.166708 | 7.880409202 |
| MaACO8_MaACO4 | 0.069128 | 0.045816 | 1.508822 | 3.786427189 |
| MaACO9_MaACO4 | 0.050405 | 0.039243 | 1.284434 | 3.243219307 |
| MaACO10_MaACO4 | 0.339869 | 0.124075 | 2.739223 | 10.25413257 |
| MaACO6_MaACO5 | 0.247923 | 0.160648 | 1.543272 | 13.27665901 |
| MaACO7_MaACO5 | 0.456047 | 0.281672 | 1.61907 | 23.27867033 |
| MaACO8_MaACO5 | 0.516217 | 0.282441 | 1.827697 | 23.34224787 |
| MaACO9_MaACO5 | 0.493542 | 0.291961 | 1.690435 | 24.12904865 |
| MaACO10_MaACO5 | 0.41189 | 0.122555 | 3.360869 | 10.12847788 |
| MaACO7_MaACO6 | 0.561789 | 0.286752 | 1.959142 | 23.69854461 |
| MaACO8_MaACO6 | 0.525712 | 0.266863 | 1.96997 | 22.05477906 |
| MaACO9_MaACO6 | 0.495144 | 0.247074 | 2.004026 | 20.41937812 |
| MaACO10_MaACO6 | 0.46804 | 0.193159 | 2.423083 | 15.9635317 |
| MaACO8_MaACO7 | 0.070942 | 0.057472 | 1.234373 | 4.749740455 |
| MaACO9_MaACO7 | 0.006321 | 0.005469 | 1.155651 | 0.45202367 |
| MaACO10_MaACO8 | 0.592984 | 0.28009 | 2.117123 | 23.14790642 |
| MaACO9_MaACO8 | 0.067433 | 0.05167 | 1.30507 | 4.270277333 |
| MaACO10_MaACO9 | 0.604304 | 0.262064 | 2.305943 | 21.65816672 |

**Table S10:** Putative functions of Cis-regulatory elements identified M. acuminata ACS promoter region.

| **Cis elements**  **(ACS)** | **Functions** |
| --- | --- |
| ABRE | cis-acting element involved in the abscisic acid responsiveness |
| BOX 4 | part of a conserved DNA module involved in light responsiveness |
| BOX111 | part of a module for light response |
| AE-box | part of a module for light response |
| CCAT- BOX | common cis-acting element in promoter and enhancer regions |
| ARE | cis-acting regulatory element essential for the anaerobic induction |
| CGTCA-motif | cis-acting regulatory element involved in the MeJA-responsiveness |
| G-box | cis-acting regulatory element involved in light responsiveness |
| GCN4_motif | cis-regulatory element involved in endosperm expression |
| LAMP-element | part of a light responsive element |
| TATC-box | cis-acting element involved in gibberellin-responsiveness |
| TATA-box | core promoter element around -30 of transcription start |
| O2-site | cis-acting regulatory element involved in zein metabolism regulation |
| MRE | MYB binding site involved in light responsiveness |
| GATA BOX | part of a light responsive element |
| TC-rich repeats | cis-acting element involved in defense and stress responsiveness |
| TCT-motif | part of a light responsive element |
| TGACG-motif | cis-acting regulatory element involved in the MeJA-responsiveness |
| GC-Motif | enhancer-like element involved in anoxic specific inducibility |
| Sp1 | light responsive element |
| TCCC-Motif | part of a light responsive element |
| ATCT-Motif | part of a conserved DNA module involved in light responsiveness |
| p-box | gibberellin-responsive element |
| TGA-Element | auxin-responsive element |
| GT 1-Motif | light responsive element |
| ATC Motif | part of a conserved DNA module involved in light responsiveness |
| CAT Box | cis-acting regulatory element related to meristem expression |
| GARE-motif | gibberellin-responsive element |
| AT-rich element | binding site of AT-rich DNA binding protein (ATBP-1) |
| LTR | cis-acting element involved in low-temperature responsiveness |
| ACE | cis-acting element involved in light responsiveness |
| AuxRR- core | cis-acting regulatory element involved in auxin responsiveness |
| CAG-motif | part of a light response element |
| HD-ZIP 1 | element involved in differentiation of the palisade mesophyll cells |
| I-Box | part of a light responsive element |
| RY-element | cis-acting regulatory element involved in seed-specific regulation |
| TCA-element | cis-acting element involved in salicylic acid responsiveness |
| ACA-motif | part of gapA in (gapA-CMA1) involved with light responsiveness |
| GA-motif | part of a light responsive element |
| GTGGC-motif | part of a light responsive element |
| ATCT-motif | part of a conserved DNA module involved in light responsiveness |
| 3-AF1 Binding site | light responsive element |
| Circadian | cis-acting regulatory element involved in circadian control |
| GATA-Motif | part of a light responsive element |
| AT1-Motif | part of a light responsive module |
| L-box | part of a light responsive element |
| sbp-CMA1c | part of a light responsive element |
| 4cl-CMA2b | light responsive element |
| chs-CMA1a | part of a light responsive element |
| MSA-Like | cis-acting element involved in cell cycle regulation |

**Table S11:** Putative functions of Cis-regulatory elements identified M. acuminata ETO promoter region.

| **Cis elements (ETO)** | **Functions** |
| --- | --- |
| ABRE | cis-acting element involved in the abscisic acid responsiveness |
| ARE | cis-acting regulatory element essential for the anaerobic induction |
| CCAAT-box | MYBHv1 binding site |
| CGTCA-motif | cis-acting regulatory element involved in the MeJA-responsiveness |
| G-box | cis-acting regulatory element involved in light responsiveness |
| GATA-motif | part of a light responsive element |
| GC-motif | enhancer-like element involved in anoxic specific inducibility |
| GCN4-motif | cis-regulatory element involved in endosperm expression |
| MRE | MYB binding site involved in light responsiveness |
| TATA-Box | core promoter element around -30 of transcription start |
| TCA-element | cis-acting element involved in salicylic acid responsiveness |
| TCCC-motif | part of a light responsive element |
| TGA-Element | auxin-responsive element |
| TGACG-motif | cis-acting regulatory element involved in the MeJA-responsiveness |
| CAAT-BOX | common cis-acting element in promoter and enhancer regions |
| LTR | cis-acting element involved in low-temperature responsiveness |
| MBS | MYB binding site involved in drought-inducibility |
| TATC-BOX | cis-acting element involved in gibberellin-responsiveness |
| A-BOX | cis-acting regulatory element |
| 3AF1-Binding site | light responsive element |
| ACE | cis-acting element involved in light responsiveness |
| AE-BOX | part of a module for light response |
| AT-Rich element | binding site of AT-rich DNA binding protein (ATBP-1) |
| GA-motif | part of a light responsive element |
| GARE-Element | gibberellin-responsive element |
| TC-Rich Repeats | cis-acting element involved in defense and stress responsiveness |
| BOX-4 | part of a conserved DNA module involved in light responsiveness |
| TCT-motif | part of a light responsive element |
| GT1-motif | light responsive element |
| BOXIII | protein binding site |
| ATCT-Motif | part of a conserved DNA module involved in light responsiveness |
| BOXII | part of a light responsive element |
| CAT-BOX | cis-acting regulatory element related to meristem expression |
| LAMP-Element | light responsive element |
| MBSI | MYB binding site involved in flavonoid biosynthetic genes regulation |
| I-BOX | part of a light responsive element |

**Table S12:** Putative functions of Cis-regulatory elements identified M. acuminata ACO promoter region.

| **Cis elements (ACO)** | **Functions** |  |
| --- | --- | --- |
| ABRE | cis-acting element involved in the abscisic acid responsiveness |  |
| CCAT- BOX | common cis-acting element in promoter and enhancer regions |  |
| ARE | cis-acting regulatory element essential for the anaerobic induction |  |
| CGTCA-motif | cis-acting regulatory element involved in the MeJA-responsiveness |  |
| TATA-box | core promoter element around -30 of transcription start |  |
| O2-site | cis-acting regulatory element involved in zein metabolism regulation |  |
| MRE | MYB binding site involved in light responsiveness |  |
| TGACG-motif | cis-acting regulatory element involved in the MeJA-responsiveness |  |
| CAT Box | cis-acting regulatory element related to meristem expression |  |

**Table S13:** miRNA ACS

| **miRNA_Acc.** | **Target_Acc.** | **miRNA_**  **start** | **miRNA_**  **end** | **Target_**  **start** | **Target_**  **end** | **miRNA_aligned_fragment** | **Target_aligned_fragment** |
| --- | --- | --- | --- | --- | --- | --- | --- |
| Mac-miR396u | MaACS6 | 1 | 24 | 274 | 297 | GUCUUCCACAGCUUUCUUGAACUG | GCUUUCAAGAAAGCUCUGGCAGAU |
| Mac-miR396u | MaACS8 | 1 | 24 | 1116 | 1139 | GUCUUCCACAGCUUUCUUGAACUG | AGGAGAGAUGGAGCUGUGGAAGAC |
| Mac-miR396a | MaACS6 | 1 | 21 | 274 | 294 | UUCCACAGCUUUCUUGAACUU | GCUUUCAAGAAAGCUCUGGCA |
| Mac-miR396d | MaACS6 | 1 | 21 | 274 | 294 | UUCCACAGCUUUCUUGAACGG | GCUUUCAAGAAAGCUCUGGCA |
| Mac-miR396e | MaACS6 | 1 | 21 | 274 | 294 | UUCCACAGCUUUCUUGAACUG | GCUUUCAAGAAAGCUCUGGCA |
| Mac-miR396f | MaACS6 | 1 | 21 | 274 | 294 | UUCCACAGCUUUCUUGAACUG | GCUUUCAAGAAAGCUCUGGCA |
| Mac-miR396g | MaACS6 | 1 | 21 | 274 | 294 | UUCCACAGCUUUCUUGAACUG | GCUUUCAAGAAAGCUCUGGCA |
| Mac-miR396h | MaACS6 | 1 | 21 | 274 | 294 | UUCCACAGCUUUCUUGAACUU | GCUUUCAAGAAAGCUCUGGCA |
| Mac-miR396i | MaACS6 | 1 | 21 | 274 | 294 | UUCCACAGCUUUCUUGAACUU | GCUUUCAAGAAAGCUCUGGCA |
| Mac-miR396j | MaACS6 | 1 | 21 | 274 | 294 | UUCCACAGCUUUCUUGAACUG | GCUUUCAAGAAAGCUCUGGCA |
| Mac-miR396l | MaACS6 | 1 | 21 | 274 | 294 | UUCCACAGCUUUCUUGAACUU | GCUUUCAAGAAAGCUCUGGCA |
| Mac-miR396m | MaACS6 | 1 | 21 | 274 | 294 | UUCCACAGCUUUCUUGAACUU | GCUUUCAAGAAAGCUCUGGCA |
| Mac-miR396n | MaACS6 | 1 | 21 | 274 | 294 | UUCCACAGCUUUCUUGAACUG | GCUUUCAAGAAAGCUCUGGCA |
| Mac-miR396o | MaACS6 | 1 | 21 | 274 | 294 | UUCCACAGCUUUCUUGAACGG | GCUUUCAAGAAAGCUCUGGCA |
| Mac-miR396t | MaACS6 | 1 | 24 | 274 | 297 | GUUUUCCACAGCUUUCUUGAACUG | GCUUUCAAGAAAGCUCUGGCAGAU |
| Mac-miR396t | MaACS14 | 1 | 24 | 1483 | 1506 | GUUUUCCACAGCUUUCUUGAACUG | CGCAUCAAGAGAGUUGUUGAUAAC |
| Mac-miR396t | MaACS8 | 1 | 24 | 1116 | 1139 | GUUUUCCACAGCUUUCUUGAACUG | AGGAGAGAUGGAGCUGUGGAAGAC |
| Mac-miRN2003a | MaACS2 | 1 | 22 | 87 | 109 | CGGAAGGAUCAUUGUCG-AGACC | GGCCUACGAUAAUGAUCCUUUCC |
| Mac-miRN2003a | MaACS3 | 1 | 22 | 102 | 124 | CGGAAGGAUCAUUGUCG-AGACC | GGCCUACGAUAAUGAUCCUUUCC |
| Mac-miRN2003b | MaACS3 | 1 | 22 | 102 | 124 | CGGAAGGAUCAUUGUCG-AGACC | GGCCUACGAUAAUGAUCCUUUCC |
| Mac-miRN2003b | MaACS2 | 1 | 22 | 87 | 109 | CGGAAGGAUCAUUGUCG-AGACC | GGCCUACGAUAAUGAUCCUUUCC |
| Mac-miRN2003c | MaACS2 | 1 | 22 | 87 | 109 | CGGAAGGAUCAUUGUCG-AGACC | GGCCUACGAUAAUGAUCCUUUCC |
| Mac-miRN2003c | MaACS3 | 1 | 22 | 102 | 124 | CGGAAGGAUCAUUGUCG-AGACC | GGCCUACGAUAAUGAUCCUUUCC |
| Mac-miR159c | Mac-miR159c | 1 | 20 | 237 | 256 | CUUGGAUUGAAGGGAGCUCC | GGAGCUCGCUUUGUUCCAGG |
| Mac-miR159f | MaACS4 | 1 | 20 | 237 | 256 | CUUGGAUUGAAGGGAGCUCC | GGAGCUCGCUUUGUUCCAGG |
| Mac-miR319a | MaACS4 | 1 | 21 | 235 | 255 | UUGGACUGAAGGGAGCUCCCU | CGGGAGCUCGCUUUGUUCCAG |
| Mac-miR319b | MaACS4 | 1 | 21 | 235 | 255 | UUGGACUGAAGGGAGCUCCCU | CGGGAGCUCGCUUUGUUCCAG |
| Mac-miR319c | MaACS4 | 1 | 21 | 235 | 255 | UUGGACUGAAGGGAGCUCCCU | CGGGAGCUCGCUUUGUUCCAG |
| Mac-miR319d | MaACS4 | 1 | 21 | 235 | 255 | UUGGACUGAAGGGAGCUCCCU | CGGGAGCUCGCUUUGUUCCAG |
| Mac-miR319e | MaACS4 | 1 | 21 | 235 | 255 | UUGGACUGAAGGGAGCUCCCU | CGGGAGCUCGCUUUGUUCCAG |
| Mac-miR319f | MaACS4 | 1 | 21 | 236 | 256 | CUUGGACUGAAGGGAGCUCCC | GGGAGCUCGCUUUGUUCCAGG |
| Mac-miR319g | MaACS4 | 1 | 20 | 236 | 255 | UUGGACUGAAGGGAGCUCCC | GGGAGCUCGCUUUGUUCCAG |
| Mac-miR319h | MaACS4 | 1 | 21 | 236 | 256 | CUUGGACUGAAGGGAGCUCCC | GGGAGCUCGCUUUGUUCCAGG |
| Mac-miR319i | MaACS4 | 1 | 21 | 236 | 256 | CUUGGACUGAAGGGAGCUCCC | GGGAGCUCGCUUUGUUCCAGG |
| Mac-miR319j | MaACS4 | 1 | 21 | 236 | 256 | CUUGGACUGAAGGGAGCUCCC | GGGAGCUCGCUUUGUUCCAGG |
| Mac-miR319k | MaACS4 | 1 | 21 | 236 | 256 | CUUGGACUGAAGGGAGCUCCC | GGGAGCUCGCUUUGUUCCAGG |
| Mac-miR396b | MaACS6 | 1 | 21 | 274 | 294 | UUCCACGGCUUUCUUGAACUG | GCUUUCAAGAAAGCUCUGGCA |
| Mac-miR396k | MaACS6 | 1 | 21 | 274 | 294 | UUCCACGGCUUUCUUGAACUG | GCUUUCAAGAAAGCUCUGGCA |
| Mac-miR396u | MaACS6 | 1 | 24 | 1116 | 1139 | GUCUUCCACAGCUUUCUUGAACUG | AGGAGAGAUGGAGCUGUGGAAGAA |
| Mac-miR396u | MaACS4 | 1 | 24 | 1116 | 1139 | GUCUUCCACAGCUUUCUUGAACUG | AGGAGAGAUGGAGCUGUGGAAGAA |
| Mac-miRN2002 | MaACS13 | 1 | 21 | 557 | 577 | UUGGAGUAGCACCGGAAGUCU | UAACUGCAGGUGCUACUCCUG |
| Mac-miRN2002 | MaACS14 | 1 | 21 | 575 | 595 | UUGGAGUAGCACCGGAAGUCU | UGACAGCUGGUGCAACUCCAG |
| Mac-miR159a | MaACS4 | 1 | 21 | 236 | 256 | UUUGGAUUGAAGGGAGCUCUA | GGGAGCUCGCUUUGUUCCAGG |
| Mac-miR159b | MaACS4 | 1 | 21 | 236 | 256 | UUUGGAUUGAAGGGAGCUCUA | GGGAGCUCGCUUUGUUCCAGG |
| Mac-miR159d | MaACS4 | 1 | 21 | 236 | 256 | UUUGGAUUGAAGGGAGCUCUA | GGGAGCUCGCUUUGUUCCAGG |
| Mac-miR159e | MaACS4 | 1 | 21 | 236 | 256 | UUUGGAUUGAAGGGAGCUCUA | GGGAGCUCGCUUUGUUCCAGG |
| Mac-miR396t | MaACS6 | 1 | 24 | 1116 | 1139 | GUUUUCCACAGCUUUCUUGAACUG | AGGAGAGAUGGAGCUGUGGAAGAA |
| Mac-miR396t | MaACS4 | 1 | 24 | 1116 | 1139 | GUUUUCCACAGCUUUCUUGAACUG | AGGAGAGAUGGAGCUGUGGAAGAA |
| Mac-miR396u | MaACS7 | 1 | 24 | 1116 | 1139 | GUCUUCCACAGCUUUCUUGAACUG | GGGGGAGAUGGAGCUGUGGAGGAA |
| Mac-miR396u | MaACS5 | 1 | 24 | 1116 | 1139 | GUCUUCCACAGCUUUCUUGAACUG | AGGAGAGAUGGAGCUGUGGAGGAA |
| Mac-miRN1993 | MaACS9 | 1 | 20 | 855 | 874 | AGGAUAUGAUGGCUUAAUUG | CAACGAGGCCGUCGUGUCCG |
| Mac-miRN2010 | MaACS4 | 1 | 21 | 1009 | 1030 | UGCGCGGUCCUGCG-CAAGAUC | AUGCUUGUCGAAGGACUGCGCA |

**Table S14:** miRNA ACO

| **miRNA acc** | **Target_**  **Acc.** | **miRNA_**  **start** | **miRNA_**  **end** | **Target_**  **start** | **Target_**  **end** | **miRNA_aligned_fragment** | **Target_aligned_fragment** |  |
| --- | --- | --- | --- | --- | --- | --- | --- | --- |
| Mac-miR390a | MaACO7 | 1 | 21 | 1222 | 1242 | AAGCUCAGGAGGGAUAGCGCC | CCUGCUAAUCCUCCUGAAUUU |  |
| Mac-miR390a | MaACO9 | 1 | 21 | 316 | 336 | AAGCUCAGGAGGGAUAGCGCC | CCUGCUAAUCCUCCUGAAUUU |  |
| Mac-miR390b | MaACO7 | 1 | 21 | 1222 | 1242 | AAGCUCAGGAGGGAUAGCGCC | CCUGCUAAUCCUCCUGAAUUU |  |
| Mac-miR390b | MaACO9 | 1 | 21 | 316 | 336 | AAGCUCAGGAGGGAUAGCGCC | CCUGCUAAUCCUCCUGAAUUU |  |
| Mac-miR390c | MaACO7 | 1 | 21 | 1222 | 1242 | AAGCUCAGGAGGGAUAGCGCC | CCUGCUAAUCCUCCUGAAUUU |  |
| Mac-miR390c | MaACO9 | 1 | 21 | 316 | 336 | AAGCUCAGGAGGGAUAGCGCC | CCUGCUAAUCCUCCUGAAUUU |  |
| Mac-miR390d | MaACO7 | 1 | 21 | 1222 | 1242 | AAGCUCAGGAGGGAUAGCGCC | CCUGCUAAUCCUCCUGAAUUU |  |
| Mac-miR390d | MaACO9 | 1 | 21 | 316 | 336 | AAGCUCAGGAGGGAUAGCGCC | CCUGCUAAUCCUCCUGAAUUU |  |
| Mac-miR390e | MaACO7 | 1 | 21 | 1222 | 1242 | AAGCUCAGGAGGGAUAGCGCC | CCUGCUAAUCCUCCUGAAUUU |  |
| Mac-miR390e | MaACO9 | 1 | 21 | 316 | 336 | AAGCUCAGGAGGGAUAGCGCC | CCUGCUAAUCCUCCUGAAUUU |  |
| Mac-miR390f | MaACO7 | 1 | 21 | 1222 | 1242 | AAGCUCAGGAGGGAUAGCGCC | CCUGCUAAUCCUCCUGAAUUU |  |
| Mac-miR390f | MaACO9 | 1 | 21 | 316 | 336 | AAGCUCAGGAGGGAUAGCGCC | CCUGCUAAUCCUCCUGAAUUU |  |
| Mac-miR391 | MaACO5 | 1 | 22 | 745 | 766 | CCGCAGGAGAGAUGAUGCCGCU | GAUGCUAUUAUUUCUCCUGCUG |  |
| Mac-miR172a | MaACO5 | 1 | 21 | 157 | 177 | AGAAUCUUGAUGAUGCUGCAU | AAGCAACAUUAUGAAGAAUCU |  |
| Mac-miR172b | MaACO5 | 1 | 21 | 157 | 177 | AGAAUCUUGAUGAUGCUGCAU | AAGCAACAUUAUGAAGAAUCU |  |
| Mac-miR172c | MaACO5 | 1 | 21 | 157 | 177 | AGAAUCUUGAUGAUGCUGCAU | AAGCAACAUUAUGAAGAAUCU |  |
| Mac-miR172d | MaACO5 | 1 | 21 | 157 | 177 | AGAAUCUUGAUGAUGCUGCAU | AAGCAACAUUAUGAAGAAUCU |  |
| Mac-miR172e | MaACO5 | 1 | 21 | 157 | 177 | AGAAUCUUGAUGAUGCUGCAC | AAGCAACAUUAUGAAGAAUCU |  |
| Mac-miR172f | MaACO5 | 1 | 21 | 157 | 177 | AGAAUCUUGAUGAUGCUGCAU | AAGCAACAUUAUGAAGAAUCU |  |
| Mac-miR172h | MaACO5 | 1 | 21 | 157 | 177 | AGAAUCUUGAUGAUGCUGCAC | AAGCAACAUUAUGAAGAAUCU |  |
| Mac-miR172i | MaACO5 | 1 | 21 | 157 | 177 | AGAAUCUUGAUGAUGCUGCAU | AAGCAACAUUAUGAAGAAUCU |  |
| Mac-miR172j | MaACO5 | 1 | 21 | 157 | 177 | AGAAUCUUGAUGAUGCUGCAU | AAGCAACAUUAUGAAGAAUCU |  |
| Mac-miR172k | MaACO5 | 1 | 21 | 157 | 177 | AGAAUCUUGAUGAUGCUGCAU | AAGCAACAUUAUGAAGAAUCU |  |
| Mac-miR172l | MaACO5 | 1 | 21 | 157 | 177 | AGAAUCUUGAUGAUGCUGCAU | AAGCAACAUUAUGAAGAAUCU |  |
| Mac-miR172m | MaACO5 | 1 | 21 | 157 | 177 | AGAAUCUUGAUGAUGCUGCAU | AAGCAACAUUAUGAAGAAUCU |  |
| Mac-miR172n | MaACO5 | 1 | 21 | 157 | 177 | AGAAUCUUGAUGAUGCUGCAU | AAGCAACAUUAUGAAGAAUCU |  |
| Mac-miR172o | MaACO5 | 1 | 21 | 157 | 177 | AGAAUCUUGAUGAUGCUGCAU | AAGCAACAUUAUGAAGAAUCU |  |
| Mac-miR172p | MaACO5 | 1 | 21 | 157 | 177 | AGAAUCUUGAUGAUGCUGCAU | AAGCAACAUUAUGAAGAAUCU |  |
| Mac-miR172q | MaACO5 | 1 | 21 | 157 | 177 | AGAAUCUUGAUGAUGCUGCAU | AAGCAACAUUAUGAAGAAUCU |  |
| Mac-miRN1999 | MaACO2 | 1 | 22 | 898 | 919 | CGGUGAUGGUAGCCGUCGGCCU | GCUAUGAAGGCUACUGUUACUG |  |
| Mac-miR172g | MaACO5 | 1 | 21 | 157 | 177 | GGAAUCUUGAUGAUGCUGCAU | AAGCAACAUUAUGAAGAAUCU |  |
| Mac-miR827 | MaACO6 | 1 | 21 | 404 | 424 | UUAGAUGACCAUCAGCAAACA | GACUUGAUGAUGAUCAUCUUA |  |

**Table S15:** miRNA ETO

| miRNA_  Acc. | Target_  Acc. | miRNA_  start | miRNA_  end | Target_  start | Target_  end | miRNA_aligned_fragment | Target_aligned_fragment |
| --- | --- | --- | --- | --- | --- | --- | --- |
| Mac-miRN2009 | MaETO2 | 1 | 21 | 2014 | 2034 | UGAGCAGCUGUUUAUGGACUU | GAGCCCAUGAAUAGCAGCUUA |
| Mac-miR171i | MaETO1 | 1 | 21 | 2064 | 2084 | CGAUUGAGCCGUGCCAAUAUC | UCUGUUGCUACGGUUGAAUUG |
| Mac-miR482a | MaETO1 | 1 | 22 | 1914 | 1935 | UUUCCAAUGCCUCCCAUGCCAA | GCAGCAUGGGAAGCAGUGGGAC |
| Mac-miR171a | MaETO1 | 1 | 21 | 2064 | 2084 | UGAUUGAGCCGUGCCAAUAUC | UCUGUUGCUACGGUUGAAUUG |
| Mac-miR171b | MaETO1 | 1 | 21 | 2064 | 2084 | UGAUUGAGCCGUGCCAAUAUC | UCUGUUGCUACGGUUGAAUUG |
| Mac-miR171d | MaETO1 | 1 | 21 | 2064 | 2084 | UGAUUGAGCCGUGCCAAUAUC | UCUGUUGCUACGGUUGAAUUG |
| Mac-miR171e | MaETO1 | 1 | 21 | 2064 | 2084 | UGAUUGAGCCGUGCCAAUAUC | UCUGUUGCUACGGUUGAAUUG |
| Mac-miR171h | MaETO1 | 1 | 21 | 2064 | 2084 | UGAUUGAGCCGUGCCAAUAUC | UCUGUUGCUACGGUUGAAUUG |
| Mac-miR171j | MaETO1 | 1 | 21 | 2064 | 2084 | UGAUUGAGCCGUGCCAAUAUC | UCUGUUGCUACGGUUGAAUUG |
| Mac-miR528 | MaETO2 | 1 | 21 | 711 | 731 | UGGAAGGGGCAUGCAGAGGAG | CUCCUCUUCCUCCUCCUUCCG |
| Mac-miRN2009 | MaETO2 | 1 | 21 | 118 | 139 | UGAGCAGCUGUUUAU-GGACUU | UCGUCCGAUAAGCCGCUGCUCC |
